# Higher rate of skin rash in a phase II trial with weekly nanoparticle albumin-bound paclitaxel and cisplatin combination in Chinese breast cancer patients

**DOI:** 10.1186/1471-2407-13-232

**Published:** 2013-05-09

**Authors:** Li Chen Tang, Bi Yun Wang, Si Sun, Jian Zhang, Zhen Jia, Yun Hua Lu, Geng Hong Di, Zhi Ming Shao, Xi Chun Hu

**Affiliations:** 1 Department of Breast Surgery, Fudan University Shanghai Cancer Center, Fudan University, Shanghai, 200032, China; 2 Department of Medical Oncology, Fudan University Shanghai Cancer Center, Fudan University, Shanghai, 200032, China; 3 Department of Oncology, Shanghai Medical College, Fudan University, Shanghai, 200032, China

**Keywords:** Phase II study, Albumin-bound paclitaxel, Cisplatin, Rash

## Abstract

**Abstracts:**

**Background**

The aim of this sub-study is to explore the incidence of skin rash among advanced breast cancer(ABC) patients in a phase II trial treated with weekly nab-paclitaxel and cisplatin combination.

**Methods**

Nab-paclitaxel(125 mg/m^2^) was administered on days 1, 8, 15, followed by cisplatin(75 mg/m^2^) on day 1 every 28 day cycle until disease progression, intolerable toxicities or the maximum of 6 cycles. Patients who received at least one injection of the study drug were included in this analysis of the incidence of skin rash among Chinese patients. Toxicity was graded using the CTCAE4.0 criteria. Statistical analysis was carried out by using SPSS 16.0 (SPSS Inc, Chicago, IL).

**Results**

Seventy three patients were enrolled and eligible for analysis. A total of 384 cycles were administered at the time of this analysis. Rash was presented in 27 patients (37.0%). The most common sites involved were face (14/27), neck (14/27), limbs (18/27) and frictional parts of the trunk (10/27). Macular and papular rash with pruritus commonly occurred 2 (95% CI: 1–7) days after the first day of chemotherapy. Only one patient developed Grade 3 skin toxicity with generalized erythroderma and disfigurement of the face requiring dose reduction. The rash gradually regressed 2 (95% CI: 1–10) days after antihistamines used, but pigmentation remained in 13/27 cases. The incidence rate of skin rash was significantly higher than what has been described for western patients (approximate 4%, P < 0.0001).

**Conclusion**

A higher rate of maculo-papular rash occurred in Chinese breast cancer patients treated with weekly nab-paclitaxel compared to western patients. The albumin component of nab-paclitaxel might be the cause of the skin disorder.

**Trial registration**

NCT01149798

## Background

Breast cancer is the most common malignancy diagnosed in women with more than 190,000 estimated new cases in USA in 2009 [1]. It is estimated that 30% of early stage patients will finally develop metastatic breast cancer (MBC) [2]. Until now, metastatic breast cancer is considered incurable. Although there are many traditional chemotherapeutic agents for the treatment of MBC, the best 5-year overall survival rate is only 20% and median survival time is between 2 to 3 years [3]. The main objectives of treatment for metastatic breast cancer are the prolongation of survival and improvement of quality of life.

In the past decade, taxane-based regimens had played an important role in the treatment of metastatic breast cancer in both adjuvant and salvage setting for patients with MBC. However, the clinical advances of taxanes have been limited by their highly hydrophobic chemical formulation and hypersensitivity. As a result, Abraxane, an albumin-bound 130-nm particle form of paclitaxel, was developed in order to avoid toxicities associated with Cremophor (BASF Corp, Ludwigshafen, Germany), the vehicle in solvent-based paclitaxel. Several clinical trials [4,5] documented the improved efficacy and favorable safety of nab-paclitaxel agent in the treatment of MBC. The phase II trial [4] confirmed that nab-paclitaxel administered every 3 weeks could improve the overall response rate to 48% for all patients and 64% for patients in first-line therapy. Time to disease progression was 26.6 weeks and 48.1 weeks for the whole population and those with confirmed tumor responses, respectively. Moreover, taxane-associated toxicities were reported to be less in frequency and severity. Another landmark phase II trial [6] demonstrated superior efficacy of weekly nab-paclitaxel compared with docetaxel, with a statistically and clinically significant prolongation of PFS (5 months) in patients receiving nab-paclitaxel 150 mg/m^2^ weekly compared with docetaxel 100 mg/m^2^ q3w. In the nab-paclitaxel 300 mg/m^2^ q3w regimen, median PFS was longer compared with docetaxel, but the superiority did not reach statistical significance. Therefore, weekly nab-paclitaxel was more desirable as previously proved in solvent-based paclitaxel [7]. Several studies exploring various polychemotherapy regimens have demonstrated improved efficacy compared to any of these agents as monotherapy [8,9].

As recently reported by Guan et al. [10], nab-paclitaxel was efficient and safe for Chinese breast cancer patients with metastastic diseases in a phase II study. However, we noticed an interesting phenomenon that in comparison with patients treated with sb-paclitaxol, those who treated with nab-patients presented rash or pruritus more frequently(9% vs. 27%, P < 0.05). This percentage was quite higher than those reported in western countries [11,12].

Therefore, in this study, we evaluated the efficacy and safety of the combination of weekly nab-paclitaxel and cisplatin in patients with advanced metastatic breast cancer. During the enrollment period, the incidence of skin rash was noted to be higher than expected. Thus, patients who received at least one injection of the study drug were included in this analysis to determine if the incidence of skin rash is indeed higher among Chinese patients in comparison with western patients.

## Methods

This is a phase II, open-label, single-institutional study at Shanghai Cancer Hospital, Fudan University. Females over 18 years with histologically confirmed invasive breast cancer who had recurrent or metastatic disease were eligible. Patients were also required to have measurable disease according to Response Evaluation Criteria In Solid Tumors (RECIST) Criteria (version 1.1) [13], good performance status (Eastern Cooperative Oncology Group performance status of 0 to 1), and a life expectancy longer than 12 weeks. Patients pretreated with taxanes could be enrolled in the trial if relapsed more than 6 months after taxanes used in neoadjuvant/adjuvant setting, or more than 3 months in metastatic disease who had documented responses when taxane administered previously. Adequate organ function was required as follows: neutrophils > 2.0*10^9^/L; platelets > 100*10^9^/L; hemoglobin > 80 g/L; serum creatinine≤upper limit of normal; bilirubin≤upper limit of normal; alkaline phosphatase <5 times of upper limit of normal; ALT/AST≤1.5 times the upper limit of the normal range except when caused by metastatic disease.

Patients were excluded if they had clinical evidence of active brain metastasis or clinically serious concurrent disease, pre-existing peripheral neuropathy more than Grade 1, concurrent hormonal or immunotherapy, or other malignancies within the last 5 years that could affect the diagnosis or assessment of breast cancer.

Nab-paclitaxel (125 mg/m^2^) was administered on days 1, 8, 15 and cisplatin (75 mg/m^2^) on day 1. Cycles were repeated every 28 days with a maximum 6 cycles unless disease progression, unacceptable toxicities, or withdrawal of consent by patient. Treatment delay was allowed up to a maximum 14 days if toxicities could not be resolved to Grade 2 orless. All patients could receive antiemetic prophylaxis per their physicians’ discretion. Routine premedication with corticosteroid or antihistamines was not used. Toxicities were observed, recorded and graded according to Common Terminology Criteria for Adverse Events (CTC AE) 4.0 [14]. Statistical analysis was carried out by using SPSS 16.0 (SPSS Inc, Chicago, IL).

This study was approved by the Ethics Committee of Cancer Hospital, Fudan University and was registered on http://www.clinicaltrials.gov (Number: NCT01149798). Our study was conducted strictly adhering to guidelines for the reporting of tumor marker studies (REMARK) [15]. Written informed consents for participation in the study and image publication were obtained from participants.

## Results

From June 2010 to October 2011, 73 patients who had received at least one injection of the study drug were enrolled and qualified for analysis. A total of 384 cycles were administered. Patients characteristics are described in Table 1.

**Table 1:** Patient characteristics (n = 73)

**Characteristic**	**No.**	**%**
Age,years		
Median	49	
Range	33-65	
Amenorrhea		
Premenopausal	29	39.7
Postmenopausal	44	60.3
Radical mastectomy		
No previous mastectomy	5	6.8
Previous mastectomy	68	93.2
Time to first relapse, years		
Median	2.55	
Range	0.4-17	
No. of metastatic sites		
1	15	20.5
2	24	32.9
3 or more	34	46.6
Metastatic sites		
Visceral	59	80.8
Lung	40	54.8
Liver	27	37.0
Nonvisceral	14	19.2
Bone	23	31.5
ER status		
Positive	40	54.8
Negative	32	43.8
Unknown	1	1.4
PR status		
Positive	33	45.2
Negative	38	52.1
Unknown	2	2.7
HER-2 status		
Positive	18	24.7
Negative	52	71.2
Unknown	3	4.1
Lines of chemotherapy		
First line	36	49.3
Second line	28	38.4
Third or more	9	12.3
Prior chemotherapy		
Adjuvant/neoadjuvant (n = 68)		
Anthracycline containing	53	72.6
Taxane containing	34	50.0
Both	27	39.7
Chemotherapy for MBC (n = 37)		
Anthracycline containing	6	16.2
Taxane containing	12	32.4
Both	3	8.1

**Figure 1:**
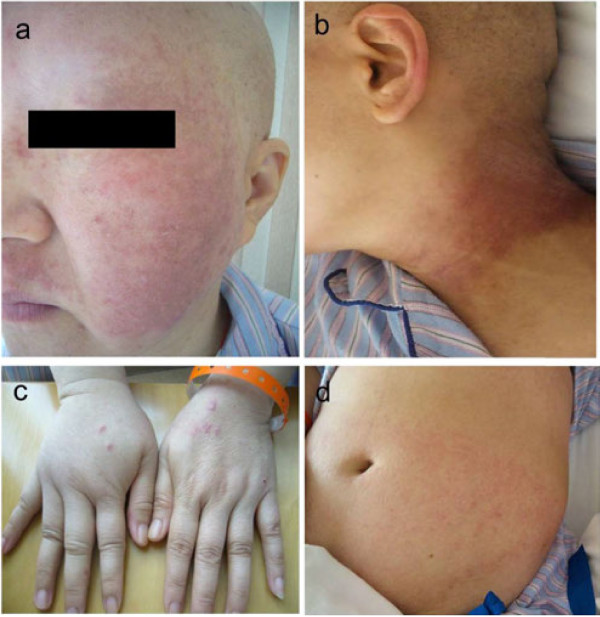
Rash and pigmentaion on face (a), neck (b), limbs (c) and abdominal wall (d).

**Figure 2:**
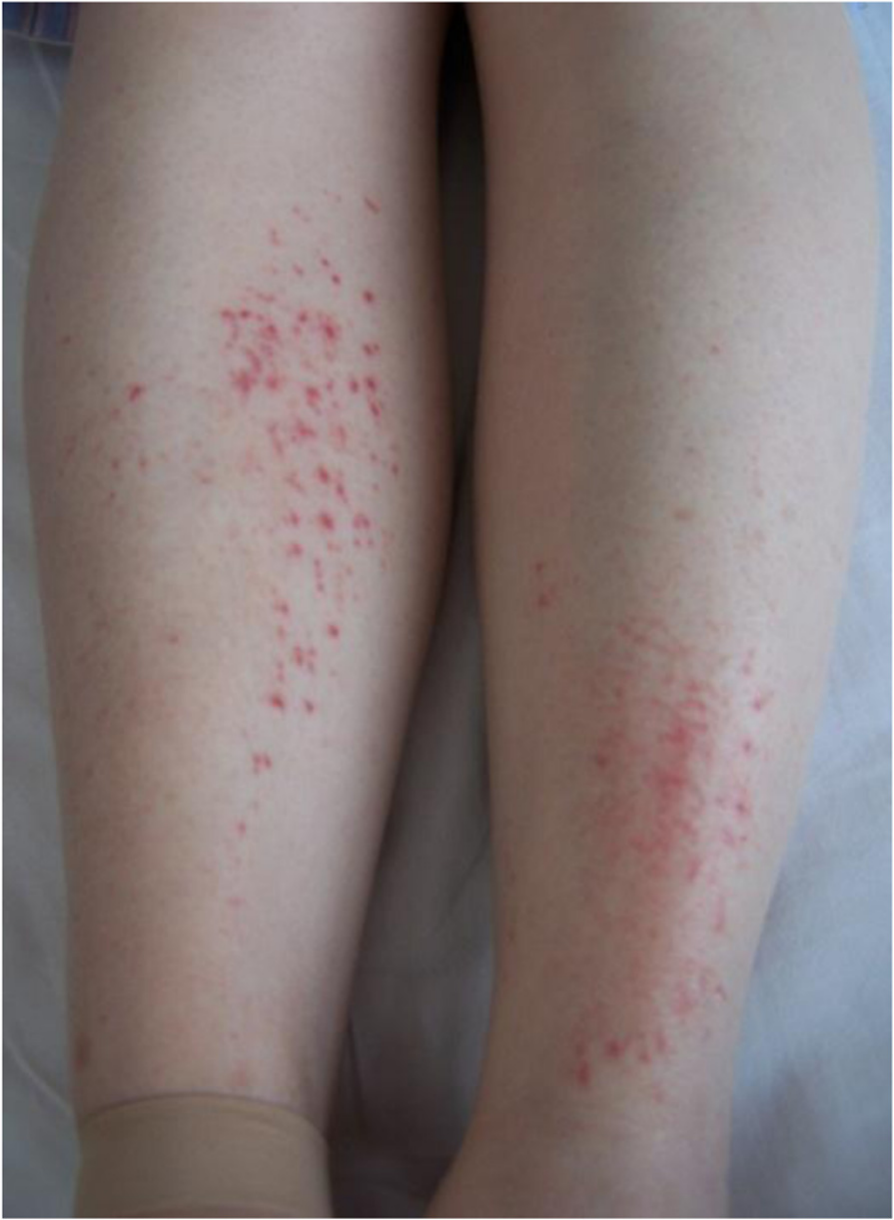
Macular rash anterior tibia.

Rash was observed in 27 patients (37.0%) among the 73 subjects since the start of their regimens, unexpectedly higher than what has been reported for western patients. The most common sites noted were face (14/27), neck (14/27), limbs (18/27) and frictional part of the trunk such as chest, abdominal wall (10/27) and haunch(3/27) (Figure 1). Rash was infrequently seen over the anterior tibia area (2/27) (Figure 2).

Macular and papular rash eruption broke out with pruritus at a median of the 2 (95% CI: 1–7) days after the infusion of nab-paclitaxel and cisplatin. The rash ocurred at a median of cycle 2, ranging from cycle 1 to 5. Nineteen of 73 patients suffered from Grade 1 maculopapular rash and 7 patients suffered from Grade 2 and all of those presented with pruritus. One subject developed Grade 3 maculopapular rash, presented with generalized erythroderma and disfigurement of the face who needed dose-reduction for continuing the regimen. Neither Grade 4 skin disorders nor exfoliative dermatitis were observed in this trial.

All patients with skin rash were treated with antihistamines for external use until the disappearance of the rash. The rash gradually regressed 2 (95% CI: 1–10) days after drug use, and residual pigmentation was observed at the site of rash in 13/27 cases, especially on the face and neck. All of the pigmentations were classified into Grade 1 (covering <10% BSA and no psychosocial impact) as defined in CTCAE 4.0 and reached the peak at the median time of 14 (7–50) days and then a plateau whose regression was observed at the earliest time of 20 days after. However, there were still pigmentation remained in 6/27 cases until the termination of this study.

No fever or other severe allergic symptoms were observed during the presence of skin rash. None of these patients experienced any skin rash during the following treatment cycle but pigmentation over the area persisted in part of the patients as previous described. No significant relationship was validated between rash and age, ER status, tumor size or other clinical-pathological features (P > 0.05, data not shown).

## Discussion

Taxanes are cell cycle-specific agents that bind with high-affinity to microtubules, stabilizing and enhancing tubulin polymerization and suppressing spindle microtubule dynamics. However, the application of paclitaxel has been a challenge as prophylactic antihistaminic agents and corticosteroids are indicated for the prevention of severe or even fatal reactions. Preclinical data suggest that cremophor may also alter the pharmacodynamics and free drug availability of paclitaxel [16].

Nanotechnology is a new field of interdisciplinary research that has expanded rapidly and widely over the past 10 years to help overcome problems in medicine [17]. Nab-paclitaxel is a novel, albumin-bound, 130 nm particle formulation of paclitaxel which is delivered in a suspension of albumin particles, free from any kind of solvent. It contains albumin (human), a derivative of human blood [18]. Based on effective donor screening and product manufacturing processes, it carries an extremely remote risk for transmission of viral diseases. However, albumin of these donor may have the antigenic effects for some patients [19]. Formulation and storage conditions are important for the immunogenicity of protein molecules. A study about IFN-a2a illustrated that the molecule became oxidized at room temperature which in turn induce an immune response [20]. It is also demonstrated by Hochuli [21] that changing to a liquid, HSA-free formulation, and recommending storage at 4°C had reduced the immunogenicity of the product. What is more, appropriate formulation of a protein product is highly important, particularly with respect to stabilization, because if this is inadequate the protein may aggregate or denature, which increases its immunogenic potential [22]. Last but not least, albumin itself was reported to result in a adverse event of rash [23].

Although there were a few studies which focused on nab-paclitaxel regimen worldwide, there was limited literature reported skin rash presentation among them. Yamamoto [11] (almost all the studies included in that review were related to nab-paclitaxel in breast cancer), only approximate 4% patients developed skin rash globally (Table 2). In our study, we found that the nab-paclitaxel and cisplatin combination was associated with a higher rate of rash compared to that of western patients as illustrated by Yamamoto (P < 0.0001, see Table 2). Similar rash rate at 26% was reported in another phase III study that compared nab-paclitaxel with Cremophor-EL-containing paclitaxel in Chinese MBC patients [10], similar to the rate reported in our study (P > 0.05, see Table 2). On the other hand, the skin rash rate was reported quite similar in Chinese patients and western patients that administered solvent-based paclitaxel [12] (P > 0.05) (See Table 2). The similarity in the skin rash rate among the Chinese population and a obviously higher skin rash incidence in comparison with western patients suggested a potential problem——a different mechanism of action may play a role in this new agent for breast cancer. One possible explanation is that the nab-paclitaxel was manufactured by Abraxis BioScience outside of China in our study. It was probable that the albumin used in this agent, which is produced locally in western countries, have exerted the antigenicity effect on the Chinese population leading to the allergic reaction.

**Table 2:** Differences in skin rash in Chinese breast cancer patients treated with albumin-bound and conventional paclitaxel

**Groups, Regimen**	**Reference**	**No. of patients**	**No rash**	**Rash**	**P value**	**P vaule**	**P value**	**P value**
Chinese, ABX + DDP	Our data	73	46	27	Ref.*	0.15	<0.0001	-
Chinese, ABX	Guan et al. [21]	104	76	28	0.15	Ref*	<0.0001	-
Chinese, PTX	Guan et al. [21]	106	96	10	<0.0001	0.001	Ref.*	-
Western, PTX	Seidman [22]	49	46	3	-	-	-	Ref.*
Western, ABX	Yamamoto [20]	229	220	9	<0.0001	0.001	-	0.49

*Ref. is the reference of the column. The P value resulted from the comparison of the group and the Ref. group.

In our study, most of the rash reaction was of non-immediate type, defined as occurring more than one hour after drug administration, manifested as erythematous macules and infiltrated papules [24]. In a recent phase I trial in NSCLC, 300 mg/m^2^ was confirmed as the MTD and Grade 3 skin rash was one of the DLTs [25].

As a limitation, the rate of skin rash in western patients treated with nab-paclitaxel is taken from the literature and not from the western patients treated in the same trial with the same regimen. The regimen interval for nab-paclitaxel was also not in accordance as reported which may cause the potential bias for statisical comparison. However, the phenomenon should be paid attention to for further study.

## Conclusion

A higher rate of maculo-papular rash occurred in Chinese breast cancer patients treated with weekly nab-paclitaxel and cisplatin. We speculate that the albumin component of nab-paclitaxel might be the cause of the skin disorder. The development of these novel agents and their incorporation to the existing treatment regimens for the management of MBC is of high priority. Efforts should focus on decreasing the side effects while improving the quality of life of the patient. Further improvements in our understanding of the pathogenesis underlying the side effects are needed in the management of these patients.

## Competing interests

No competing interests declared.

## Authors’ contributions

LCT participated in the data collection, carried out the statistical analysis and drafted the manuscript. XCH and BYW conceived of the study, and participated in its design and coordination and helped to draft the manuscript. SS participated in the data check. GHD and ZMS provided advice to improve the study. YHL, JZ and ZJ participated in the data collection. All authors read and approved the final manuscript.

## Pre-publication history

The pre-publication history for this paper can be accessed here:

http://www.biomedcentral.com/1471-2407/13/232/prepub
